# Integrated Transcriptomic and Metabolomic Analysis Reveals Molecular Signatures Associated with Natural Degeneration of *Puccinia striiformis* f. sp. *tritici*

**DOI:** 10.3390/cimb48020169

**Published:** 2026-02-02

**Authors:** Congying Yuan, Tianyu Long, Jiani Dong, Bingyu Yan, Tingxuan Chen, Yubin Zhang, Yuanhan Yan, Mengyu Cheng, Sitong Xue

**Affiliations:** School of Life Science, Luoyang Normal University, 6 Jiqing Road, Luoyang 471934, China; lty_shengk123@163.com (T.L.); 13949262187@163.com (J.D.); 15290860964@139.com (B.Y.); 15893899603@163.com (T.C.); 17538977861@163.com (Y.Z.); h1525760aaaa@163.com (Y.Y.); 17630752882@163.com (M.C.); 15538932663@163.com (S.X.)

**Keywords:** wheat stripe rust, transcriptome, metabolome, natural degeneration, differentially expressed genes

## Abstract

Stripe rust of wheat, caused by the obligate biotrophic fungus *Puccinia striiformis* f. sp. *tritici* (Pst), is a devastating disease. The natural degeneration and viability loss of Pst urediniospores directly impact its dispersal and epidemic potential, yet the underlying molecular mechanisms remain unclear. This study aimed to systematically decipher the key molecular changes during the natural degeneration of Pst urediniospores using a multi-omics approach. We performed integrated transcriptomic (RNA-seq) and metabolomic (LC-MS) analyses on relatively purified fresh urediniospores (CC group) and those undergoing room-temperature-induced degeneration (CM group) of the prevalent Pst race CYR34. A total of 1622 differentially expressed genes (DEGs) and 382 differentially accumulated metabolites (DAMs) were identified. Transcriptomic analysis revealed significant downregulation of core energy and biosynthetic pathways, including ribosome biogenesis and oxidative phosphorylation. Metabolomic profiling showed that lipids and lipid-like molecules, along with organic acids and derivatives, constituted the major classes of altered metabolites. DAMs were primarily enriched in pathways such as “Metabolic pathways” and “ABC transporters.” Integrated analysis indicated a prevalent negative correlation pattern between gene expression levels and metabolite abundance. This study provides a systematic molecular landscape associated with Pst urediniospore degeneration, revealing characteristics concomitant with the suppression of energy metabolism and translation functions, thereby offering novel insights and a data foundation for understanding the mechanisms of viability maintenance and loss.

## 1. Introduction

Wheat (*Triticum aestivum* L.) is one of the most crucial global food crops, and its stable yield is closely linked to food security [1]. However, stripe rust, caused by the obligate biotrophic fungus Pst, is a devastating disease threatening wheat production [2]. As an air-borne disease, it is challenging to control [3]. The primary control strategy relies on cultivating resistant varieties, supplemented by chemical control and agricultural practices [4]. However, the widespread deployment of resistant wheat varieties readily exerts selective pressure on the Pst population, leading to the emergence of new virulent Pst races and the subsequent loss of varietal resistance [5]. Domestic and international production practices and research indicate that under normal conditions, wheat varieties with race-specific resistance typically lose their resistance to Pst within 3–5 years of large-scale cultivation. Moreover, cases exist where new varieties lose resistance even before widespread deployment [6,7]. Since the 1980s, extensive chemical control strategies have been used. While offering immediate effects and enabling short-term control of large-scale outbreaks, chemical control poses significant risks to human health and the environment [8]. Therefore, elucidating the pathogenic mechanisms of Pst remains fundamental for developing sustainable control strategies, with breeding for broad-spectrum and durable resistance being the most economical and effective long-term approach.

The wheat stripe rust pathogen relies on urediniospores for long-distance dispersal through atmospheric currents to initiate epidemics. However, under natural conditions, the viability of urediniospores is limited; they can lose infectivity within several weeks at room temperature, though viability can be prolonged under cold, dry storage [9]. The Chinese Pst race CYR34, which emerged in 2009 and was formally designated in 2016, exhibits strong virulence and has become prevalent [10]. Despite its importance, the molecular mechanisms underlying the maintenance and loss of urediniospore viability—a process critical for understanding disease cycle interruptions and developing novel control tactics—are poorly understood. Most molecular studies on Pst have focused on host–pathogen interactions during infection, utilizing techniques such as transcriptomics to dissect wheat resistance mechanisms [11] or susceptibility responses [12], and to characterize effector functions [13]. Furthermore, temporal dynamics of gene expression during infection have been documented [14]. In contrast, studies investigating the intrinsic biological processes of Pst spores themselves, particularly the systematic molecular events associated with their natural degeneration and viability loss, are scarce.

Recent advances in our molecular understanding of Pst have been facilitated by genomic resources. Initial genome sequencing projects of various Pst races revealed considerable variation in genome size (ranging from ~64 to ~130 Mb) and gene content, highlighting its genetic complexity [15,16,17,18]. For this study, we employed the high-quality reference genome of race Pst-134E36 (GCF_021901695.1) as the mapping reference. Integrated transcriptomic and metabolomic analysis provides a powerful systems biology approach to decipher regulatory networks from genes to metabolites [19]. In the context of Pst, transcriptomics has been applied to compare gene expression across different spore stages [20]. However, a comprehensive multi-omics investigation targeting the process of Pst urediniospore degeneration is still lacking.

Therefore, to fill this knowledge gap and establish a molecular framework for understanding Pst spore biology, this study employed an integrated transcriptomic and metabolomic approach. We systematically profiled the molecular changes in the prevalent Pst race CYR34 by comparing fresh, viable urediniospores with those that had undergone natural degeneration at room temperature. The objectives were to identify key differentially expressed genes and altered metabolites associated with spore degeneration; elucidate the major biological pathways and processes impacted during this process; and explore the correlations between transcriptional and metabolic changes. We hypothesize that the loss of spore viability is accompanied by a coordinated downregulation of core metabolic and biosynthetic pathways. The findings from this study will provide novel insights into the molecular basis of Pst spore longevity and degeneration, offering potential targets for strategies aimed at reducing pathogen survival and persistence.

## 2. Materials and Methods

### 2.1. Cultivation and Collection of Pst CYR34

The highly virulent Chinese Pst race CYR34, maintained in liquid nitrogen at the laboratory of Luoyang Normal University, was used in this study. Sealed glass tubes containing preserved Pst CYR34 were immersed in a 50 °C water bath for 2 min to activate the strain. The activated spores concentration used was 20–30 mg mixed with talcum powder at a 1:20 ratio. The mixture was then evenly applied onto the second leaves of 8- to 10-day-old wheat seedlings of the stripe rust-susceptible variety Avocet S (AVS) using cotton swabs. Immediately after inoculation, the plants were misted with water to form a uniform thin layer of water droplets. The inoculated plants were then placed in a dew chamber for 24 h (darkness, 10 °C, RH = 100%). After the dew period, the plants were covered with translucent plastic covers and transferred to a disease development chamber with a 16 h light (20 °C)/8 h dark (4 °C) cycle. After 17–20 days, when spores fully covered the inoculated leaves, spores were collected. The freshly collected spores were inoculated onto wheat differential hosts to identify and confirm the race. After verification, the fresh spores were re-inoculated onto the susceptible variety AVS for mass multiplication to obtain sufficient spore quantity. The obtained spores were divided into two experimental groups: one group was flash-frozen in liquid nitrogen and stored at −80 °C (control group, comprising what was named CC1, CC2, CC3), and the urediniospores in the experimental group were placed in a constant temperature and humidity incubator at 25 °C and 20% relative humidity to simulate an ambient dry environment for a 30-day aging treatment. After treatment, some spores were flash-frozen in liquid nitrogen and then transferred to a −80 °C freezer for long-term storage for later use; the remaining spores were immediately inoculated onto seedlings of the susceptible wheat cultivar AVS at the two-leaf-one-heart stage. Disease assessment was performed 15-days after inoculation according to the 0–9 disease severity rating scale. Spores with a severity rating of 0 (no visible infection symptoms) were considered inactive. Thereafter, these inactive spores, along with the untreated control group spores, were used in subsequent experiments. (treatment group, comprising what were named CM1, CM2, CM3).

### 2.2. Transcriptome Sequencing Analysis

#### 2.2.1. Total RNA Extraction, Library Construction, and Sequencing of Pst

To minimize host tissue contamination, spores were carefully collected by gently brushing the leaf surface and then passed sequentially through multiple layers of sterile gauze and a fine-mesh nylon sieve (200 mesh). Relatively purified Pst urediniospore samples were ultimately obtained. Total RNA was extracted from approximately 50 mg of the purified spore samples (CC and CM groups) using TRIzol^®^ Reagent (Invitrogen, Carlsbad, CA, USA) according to the manufacturer’s protocol. Residual genomic DNA was removed using DNase I (Takara, Otsu, Japan). RNA integrity and concentration were assessed using an Agilent 2100 Bioanalyzer (Agilent Technologies, Santa Clara, CA, USA) and a Qubit Fluorometer (Thermo Fisher Scientific, Waltham, MA, USA), respectively. Only high-quality RNA samples (RNA Integrity Number, RIN ≥ 8.0) were used for subsequent library construction. Ribosomal RNA (rRNA) was depleted from total RNA using the Ribo-Zero™ rRNA Removal Kit (Epicentre, Madison, WI, USA) to enrich for mRNA and other non-rRNA transcripts. Sequencing libraries were constructed using the NEBNext^®^ Ultra™ II Directional RNA Library Prep Kit for Illumina^®^ (NEB, Ipswich, MA, USA) following the manufacturer’s instructions. Briefly, the rRNA-depleted RNA was fragmented, followed by first-strand and second-strand cDNA synthesis. The cDNA fragments were end-repaired, adenylated, ligated with Illumina adapters, and PCR-amplified. The final libraries were quantified and their size distribution was validated using an Agilent 2100 Bioanalyzer. Paired-end sequencing (2 × 150 bp) was performed on an Illumina NovaSeq 6000 platform by Personalbio Gene Company (Shanghai, China).

To assess and exclude plant RNA contamination, RT-qPCR was performed to detect wheat housekeeping genes (TaEF-1α). No significant wheat gene expression signal was detected in any spore sample (Ct > 35), confirming minimal plant RNA contamination that would not interfere with subsequent Pst transcriptome analysis.

#### 2.2.2. Data Filtering, Assembly, Functional Annotation, and Classification

Raw sequencing reads were subjected to quality control using FastQC (v0.11.9). Adapter sequences and low-quality reads were trimmed using Trimmomatic (v0.39) with the parameters: LEADING:3, TRAILING:3, SLIDINGWINDOW:4:15, MINLEN:36. Clean reads from each sample were aligned to the *Puccinia striiformis* f. sp. *tritici* reference genome (race Pst-134E36, GenBank assembly accession: GCF_021901695.1, https://www.ncbi.nlm.nih.gov/assembly/GCF_021901695.1, accessed on 26 January 2026) using the splice-aware aligner HISAT2 (v2.2.1). To estimate the potential contamination from wheat (*Triticum aestivum*) host tissue, the unaligned reads were subsequently mapped to the wheat reference genome (IWGSC RefSeq v2.1) using Bowtie2 (v2.4.5). The percentage of reads mapping uniquely to the Pst genome exceeded 85% for all samples, while the percentage mapping to the wheat genome was consistently below 5%, indicating successful enrichment for Pst transcripts. Gene expression levels were quantified as read counts using featureCounts (v2.0.3) from the Subread package, based on the Pst genome annotation. Transcript abundance was normalized and reported as Transcripts Per Million (TPM).

#### 2.2.3. Differential Gene Screening and Enrichment Analysis

Differential gene expression analysis between the CM and CC groups was performed using the DESeq2 package (v1.38.3) in R (v 4.5.2), which models raw read counts using a negative binomial distribution [21]. Genes with an absolute value of log2 fold change (|log2FC|) ≥ 1 and a false discovery rate (FDR)-adjusted *p*-value (padj) < 0.05 were considered significantly differentially expressed genes (DEGs). Gene Ontology (GO) enrichment analysis for the identified DEGs was conducted using the clusterProfiler package (v4.10.0). GO terms with a padj < 0.05 were considered significantly enriched. Kyoto Encyclopedia of Genes and Genomes (KEGG) pathway enrichment analysis was similarly performed using clusterProfiler. The significance threshold was also set at padj < 0.05. Visualization of GO terms and KEGG pathways was performed using ggplot2 (v3.4.4).

### 2.3. Metabolomic Analysis

#### 2.3.1. Metabolite Extraction, Chromatography, Mass Spectrometry, and Metabolite Search

Metabolites were extracted from approximately 10 mg of each spore sample (CC and CM groups). Samples were homogenized in 1 mL of pre-cooled extraction solvent (methanol/acetonitrile/water, 2:2:1, *v*/*v*/*v*) using a ball mill, followed by vortexing and sonication on ice. The homogenate was incubated at −20 °C for 1 h and then centrifuged at 14,000× *g* for 15 min at 4 °C. The supernatant was collected, vacuum-dried, and reconstituted in 100 μL of acetonitrile/water (1:1, *v*/*v*) for LC-MS/MS analysis. A mixture of isotope-labeled internal standards (^13^C-glucose) was added during extraction to correct for variability due to recovery rates, ionization efficiency, and instrument drift.

Metabolomic profiling was performed using an ultra-high-performance liquid chromatography (UHPLC) system (Vanquish, Thermo Fisher Scientific) coupled with a high-resolution Q Exactive™ HF-X mass spectrometer (Thermo Fisher Scientific). Chromatographic separation was achieved on a Hypesil Gold column (C18, 100 × 2.1 mm, 1.9 μm) maintained at 40 °C. The mobile phase consisted of water with 0.1% formic acid (A) and acetonitrile with 0.1% formic acid (B). The elution gradient was as follows: 0–1 min, 2% B; 1–8 min, 2–50% B; 8–12 min, 50–98% B; 12–14 min, 98% B; 14–14.1 min, 98–2% B; 14.1–16 min, 2% B. The flow rate was 0.3 mL/min. Mass spectrometry data were acquired in both positive and negative ion modes with a full scan range of *m*/*z* 70–1050. Data-dependent acquisition (DDA) mode was used to obtain MS/MS spectra for metabolite identification.

A pooled quality control (QC) sample was prepared by mixing equal volumes of all individual sample extracts. This QC sample was injected at the beginning of the sequence for column conditioning and repeatedly analyzed after every four experimental samples throughout the analytical run to monitor system stability.

Metabolite identification was performed through multi-dimensional matching: (1) exact mass measurement with mass error < 5 ppm; (2) retention time index alignment; (3) MS/MS fragment pattern matching against established spectral libraries including HMDB (Human Metabolome Database), MassBank, and an in-house standard library (match score threshold > 80%); and (4) comparison with authentic chemical standards when available.

#### 2.3.2. Metabolite Identification, Differential Comparison, and Enrichment Analysis

Raw LC-MS data were processed using the Compound Discoverer software (v3.3, Thermo Fisher Scientific) for peak picking, alignment, and retention time correction. Metabolites were identified by matching the acquired MS/MS spectra against public databases (mzCloud, HMDB, MassBank) and a custom in-house spectral library with a mass tolerance of 5 ppm for precursor ions and 10 ppm for fragment ions. The identification confidence followed the reporting standards proposed by the Metabolomics Standards Initiative (MSI). Identifications supported by MS/MS spectral matching (Level 2 or Level 1) were retained for subsequent analysis. For relative quantification, peak areas were normalized to the total peak area of the QC samples (Probabilistic Quotient Normalization). Multivariate statistical analysis, specifically Orthogonal Partial Least Squares-Discriminant Analysis (OPLS-DA), was performed using SIMCA-P software (v16.0.2, Umetrics) to discriminate between the CC and CM groups. The Variable Importance in Projection (VIP) score from the OPLS-DA model was used to rank the contribution of each metabolite to group separation. Differential metabolites (DAMs) were screened based on the following criteria: VIP > 1.0 (from the OPLS-DA model), |log2FC| ≥ 1, and *p*-value < 0.05 (from a two-tailed Student’s *t*-test). Enriched KEGG pathways for the DAMs were identified using the MetaboAnalyst 5.0 web server, with a significance threshold of padj < 0.05.

### 2.4. Integrated Transcriptomic and Metabolomic Analysis

To explore the correlations between transcriptional and metabolic changes, an integrated analysis was performed. First, KEGG pathway co-enrichment analysis was conducted to identify biological pathways that were significantly altered in both the transcriptome and metabolome datasets. Pathways with padj < 0.05 in both analyses were considered co-enriched.

Subsequently, a correlation network analysis was performed to identify significant gene–metabolite pairs. For this analysis, we focused on DEGs annotated as enzymes (EC numbers) in the KEGG database and all identified DAMs. The corresponding enzyme-encoding genes for each metabolite were retrieved based on KEGG reaction and pathway maps. Pearson correlation coefficients (r) were calculated between the TPM values of these genes and the relative abundance of their corresponding metabolites across all six biological samples (CC1-3, CM1-3). Significant gene–metabolite associations were filtered using a stringent threshold of |r| > 0.85 and a *p*-value < 0.01 [22]. The resulting network was visualized using the igraph package (v2.0.3) in R, employing a force-directed layout algorithm (Fruchterman-Reingold).

### 2.5. Gene Expression Level Validation by qRT-PCR

To technically validate the RNA-seq results, eight DEGs were selected for qRT-PCR analysis. The selection was based on two criteria: (1) their involvement in the most significantly enriched KEGG pathways and (2) a high magnitude of expression change (|log2FC| > 4) in the RNA-seq data. Gene-specific primers were designed using Primer Premier 6.0 (Premier Biosoft, Biosoft, Palo Alto, CA, USA) and are listed in Supplementary Table S1.

First-strand cDNA was synthesized from 1 μg of total RNA (the same RNA used for sequencing) using the PrimeScript™ RT reagent Kit with gDNA Eraser (Takara, Japan). qRT-PCR was performed in triplicate for each biological replicate using TB Green^®^ Premix Ex Taq™ II (Takara, Otsu, Japan) on a QuantStudio 5 Real-Time PCR System (Applied Biosystems, Waltham, MA, USA). The Pst actin gene (Gene ID: Pst134EA_028104(nei)) was used as the internal reference for normalization. Relative gene expression levels were calculated using the 2−ΔΔCt method. The correlation between the log2FC values obtained from RNA-seq and qRT-PCR was assessed using Pearson correlation analysis.

## 3. Results

### 3.1. Transcriptome Analysis

#### 3.1.1. Transcriptome Sequencing Results

The raw sequencing reads have been submitted to the NCBI Sequence Read Archive (SRA) and are available under the SRA Study accession PRJNA1374178. This study used the Illumina platform to sequence six samples (control and treatment groups). Total bases ranged from 5.9 to 7.6 Gb, meeting common sequencing depth for fungal genomes. Q30 (%) values were all above 95% (95.69~95.91%), indicating a sequencing error rate below 0.1% and high data reliability. All samples had Q20(%) > 98.88%, further confirming high quality. N (%) (ambiguous base ratio) was approximately 0.11%, indicating accurate base calling and good instrument signal interpretation (Table 1). The reference genomic source was https://ftp.ncbi.nlm.nih.gov/genomes/all/GCF/021/901/695/GCF_021901695.1_Pst134E36_v1_pri (accessed on 26 January 2026). In summary, data quality was qualified and deemed suitable for subsequent analysis.

#### 3.1.2. Differential Gene Expression Analysis

**Figure 1:**
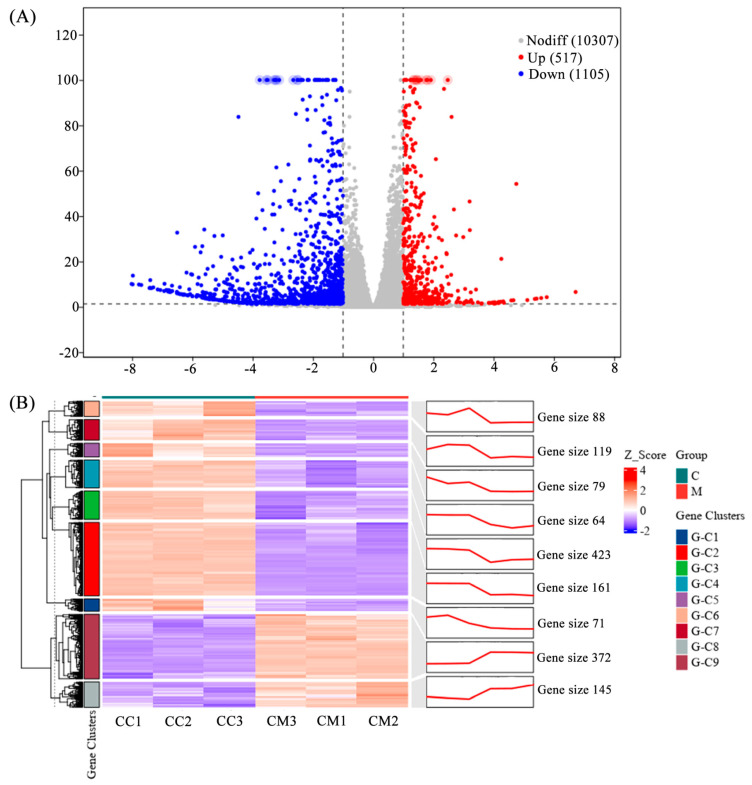
Volcano plot and cluster, and trend analysis of the transcriptome DEGs. (**A**) Volcano plot of the transcriptome DEGs. DEGs were identified by filtering the two-fold upregulated and downregulated genes with FDR ≤ 0.05. (**B**) Cluster and trend analysis of the transcriptome DEGs.

We performed statistical analysis of DEGs after the treatment (natural degeneration). A volcano plot shows the overall distribution of DEGs (Figure 1A). A total of 1622 DEGs were identified, comprising 517 upregulated and 1105 downregulated genes. This indicates that the expression of many genes in Pst was significantly induced or inhibited by the treatment. Cluster and trend analysis (Figure 1B) showed that DEGs were divided into nine clusters. Among these, seven clusters showed a decreasing trend in expression in the treatment group compared to the control, while two clusters showed an increasing trend.

#### 3.1.3. GO Enrichment of DEGs

**Figure 2:**
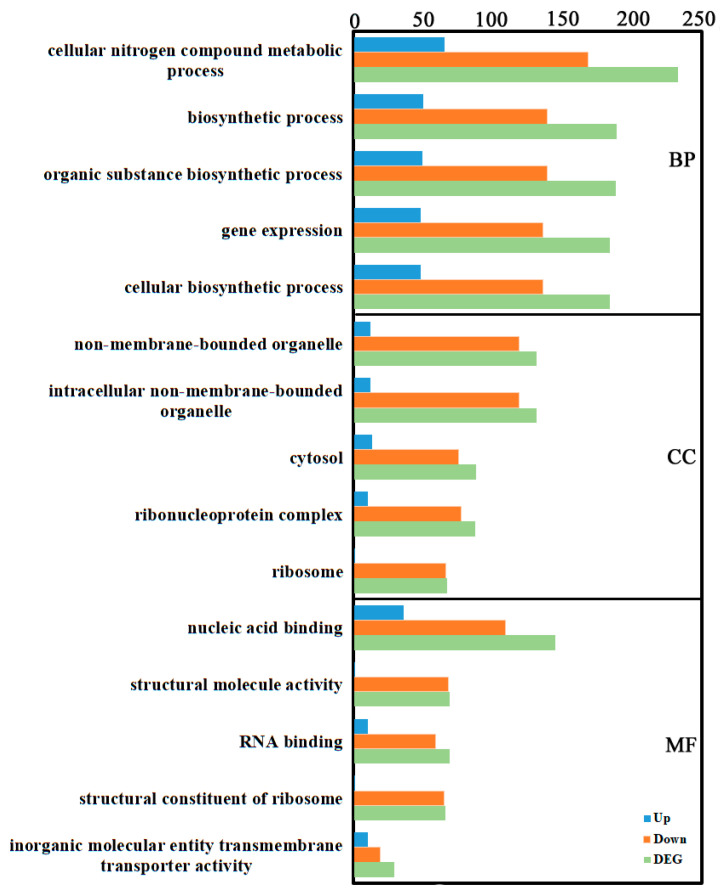
GO annotation of the transcriptome DEGs. GO terms were identified by filtering with *p*-value ≤ 0.05. The top five significant enrichments of biological processes, cellular components, and molecular functions were obtained.

Gene Ontology (GO) is a widely used bioinformatics ontology. GO includes three main categories: biological process (BP), cellular component (CC), and molecular function (MF). GO enrichment analysis of the identified DEGs revealed the following: In BP, “cellular nitrogen compound metabolic process” contained the most DEGs (233: 65 up, 168 down), followed by “biosynthetic process” (189: 50 up, 139 down). In CC, “non-membrane-bounded organelle” and “intracellular non-membrane-bounded organelle” contained the most DEGs (both 131: 12 up, 119 down), followed by “cytosol” (88: 13 up, 75 down). In MF, “nucleic acid binding” contained the most DEGs (145: 36 up, 109 down), followed by “structural molecule activity” and “RNA binding” (both 69; structural: 1 up, 68 down; RNA binding: 10 up, 59 down) (Figure 2, Supplementary Table S2).

#### 3.1.4. KEGG Enrichment of DEGs

**Figure 3:**
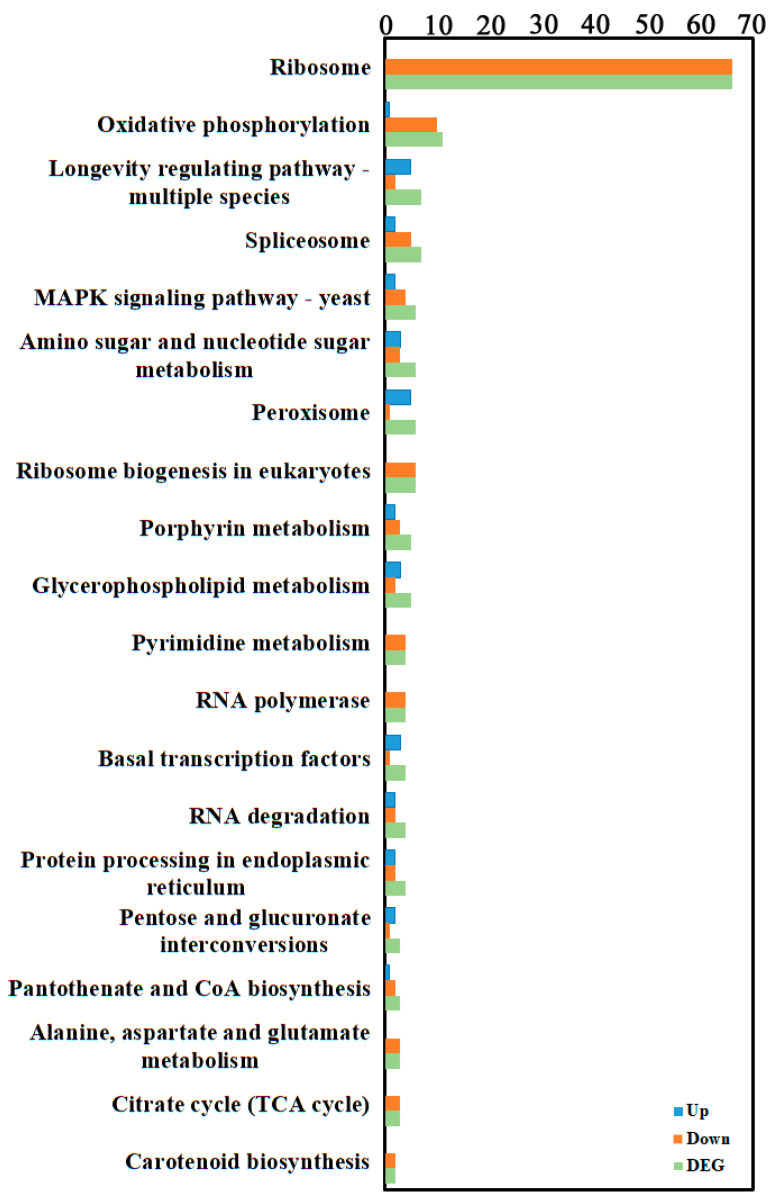
KEGG enrichment of the transcriptome DEGs. KEGG enrichment of was identified by filtering with *p*-value ≤ 0.05. The top 20 significant enrichment of the pathways were obtained.

To further investigate gene functions, genes were mapped to KEGG reference pathways, primarily belonging to Genetic Information Processing, Metabolism, and Organismal Systems branches (Figure 3, Supplementary Table S3). KEGG enrichment analysis of DEGs annotated them to 71 biological pathways, mainly involving ribosome (for example, Pst134EA_025921 corresponds to the 40S ribosomal protein S4, and Pst134EA_015752 corresponds to the 60S ribosomal protein L13E (CIMG_08619)) and oxidative phosphorylation (for example, Pst134EA_009086 corresponds to NADH dehydrogenase (ubiquinone) Fe-S protein 1, and Pst134EA_019065 corresponds to succinate dehydrogenase [ubiquinone] cytochrome b small subunit).

### 3.2. Metabolomic Analysis

#### 3.2.1. Metabolomics Quality Control

To obtain reliable and high-quality metabolomic data, quality control (QC) is essential. In this study, we used QC samples during LC-MS analysis for quality control. System stability was evaluated using QC sample chromatogram overlay, QC sample correlation analysis, and sample PCA analysis. QC chromatogram overlay showed largely overlapping peak response intensities and retention times, indicating minor variation caused by instrument error throughout the experiment (Supplementary Figure S1). Pearson correlation analysis of QC samples showed correlation coefficients close to 1, indicating high similarity in expression patterns among QC samples (Supplementary Figure S2). PCA analysis showed QC samples clustering closely together, indicating data reliability (Supplementary Figure S3). In summary, data quality was qualified, suitable for subsequent analysis.

#### 3.2.2. Metabolite Identification and Analysis

**Figure 4:**
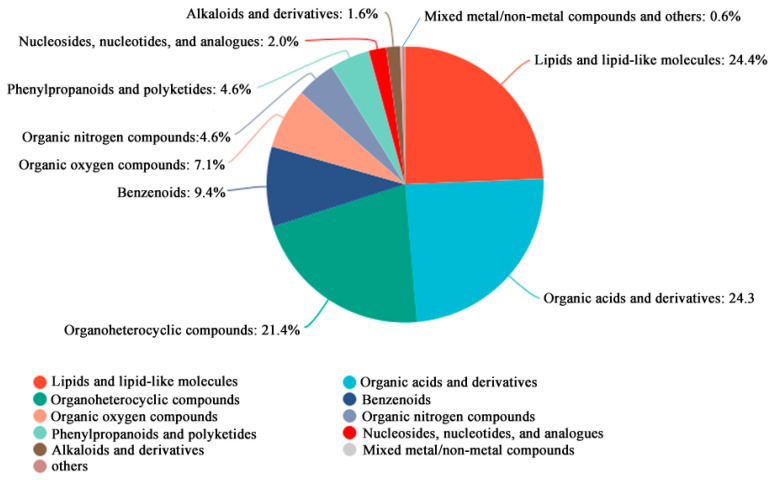
Metabolite identification analysis of metabolomics DEGs. The top 10 categories are displayed.

All identified metabolites were classified and statistically summarized based on their chemical taxonomy, displayed in a pie chart (Figure 4). Results showed the top three chemical classes by proportion were lipids and lipid-like molecules (24.4%), organic acids and derivatives (24.3%), and organoheterocyclic compounds (21.4%).

#### 3.2.3. Differential Metabolite Enrichment Analysis

**Figure 5:**
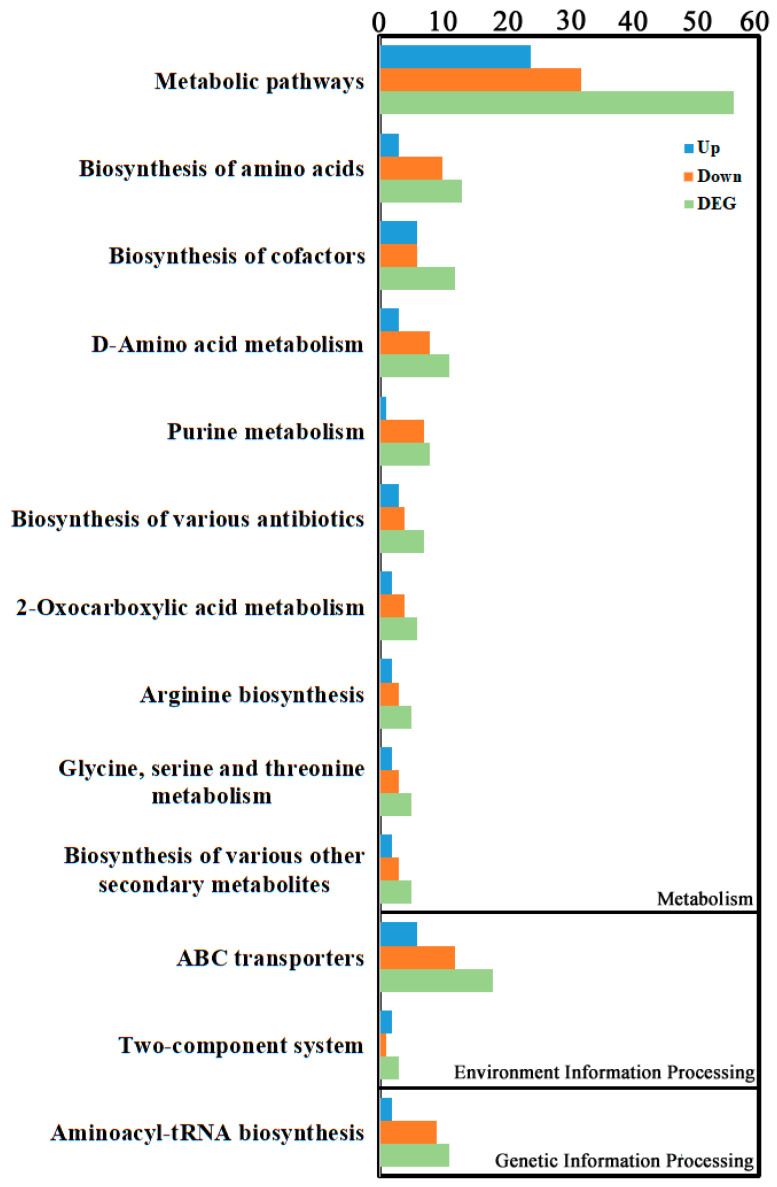
KEGG enrichment analysis of the metabolite DEGs. KEGG enrichment was identified by filtering with *p*-value ≤ 0.05. The top 10 significant enrichments of the pathways were obtained.

Enrichment classification of differential metabolites indicated they were primarily concentrated in Environmental Information Processing, Genetic Information Processing, and Metabolism categories (Figure 5, Supplementary Table S4). The top three enriched pathways were “Metabolic pathways” within Metabolism (56 differential metabolites: 24 up, 32 down), followed by “ABC transporters” within Environmental Information Processing (18 differential metabolites: 6 up, 12 down), and then “Biosynthesis of amino acids” within Metabolism (13 differential metabolites: 3 up, 10 down).

### 3.3. Integrated Transcriptomic and Metabolomic Analysis

**Figure 6:**
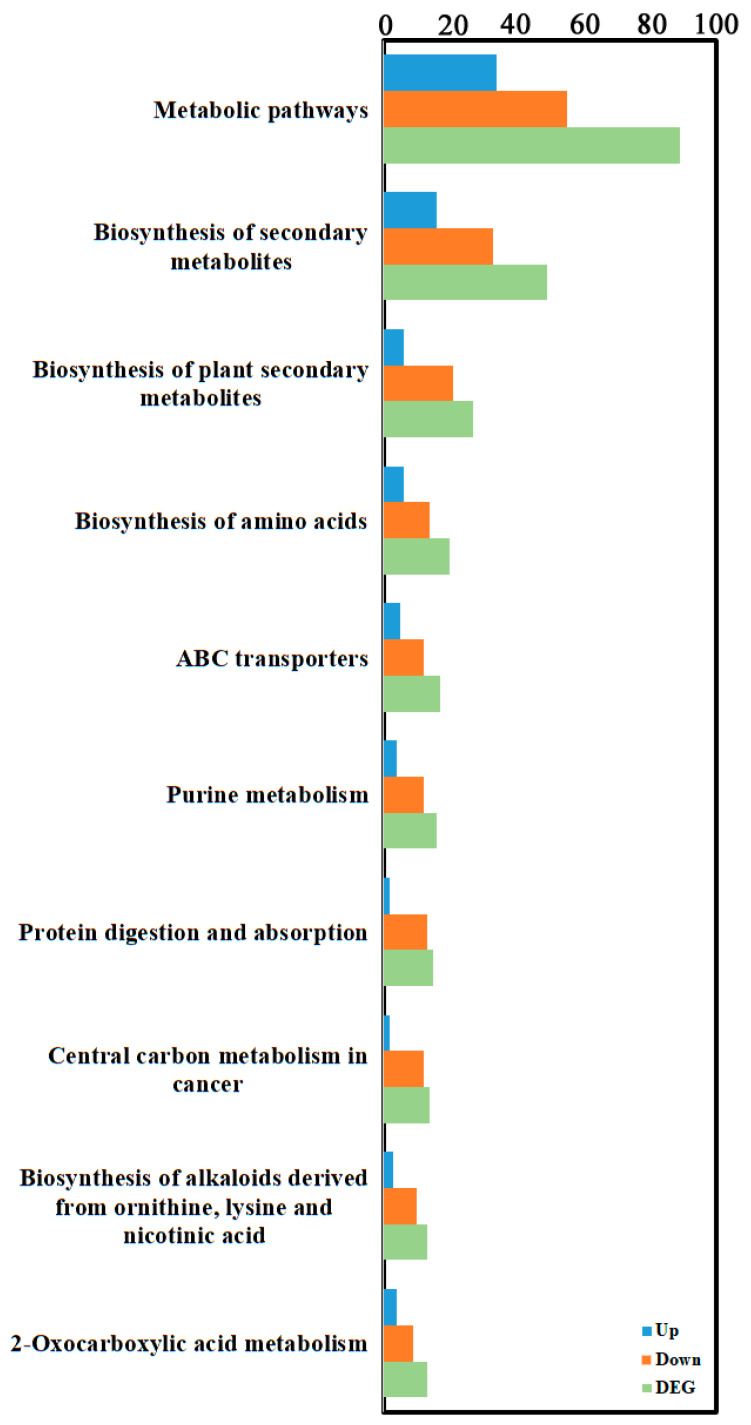
KEGG enrichment analysis of transcriptomic and metabolomic DEGs. KEGG enrichment was identified by filtering with *p*-value ≤ 0.05. The top 10 significant enrichments of the pathways were obtained.

**Figure 7:**
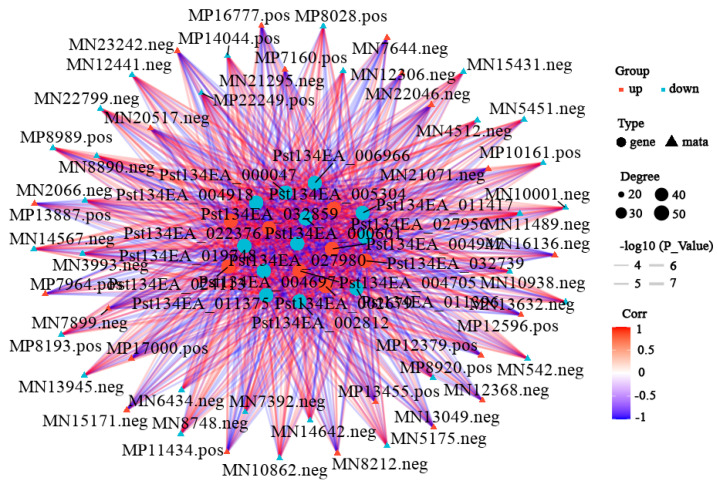
Correlation network analysis of transcriptomic and metabolomic DEGs. Correlation network analysis was identified by filtering with *p*-value ≤ 0.05. The top 20 were obtained. The color of the lines represents the magnitude of the correlation coefficient. The darker the color, the higher the positive correlation between the gene and the metabolite; conversely, the bluer the color, the stronger the negative correlation. The thickness represents the size of the *p*-value. The shape of the nodes represents the gene and metabolite, and the color of the nodes indicates the upregulation or downregulation of the gene/metabolite, with red indicating upregulation and blue indicating downregulation.

To further explore key differential genes and metabolites between the treatment and control groups, an integrated analysis of transcriptome and metabolome data was performed. Data showed 517 upregulated and 1105 downregulated differentially expressed genes, and 402 upregulated and 240 downregulated differential metabolites in the differential results. KEGG enrichment analysis (Figure 6, Supplementary Table S5) showed the most significant common pathways were metabolism-related pathways: “Metabolic pathways” (89 differential metabolites: 34 up, 55 down); followed by “Biosynthesis of secondary metabolites” (49 differential metabolites: 16 up, 33 down); and then “Biosynthesis of amino acids” (27 differential metabolites: 6 up, 21 down). Correlation network analysis revealed gene–metabolite correlations (Figure 7, Supplementary Table S6). Results indicated that most gene–metabolite pairs showed a negative association (i.e., when gene expression was downregulated, the corresponding metabolite tended to accumulate, and vice versa), while a minority showed a positive association. This co-variation pattern suggests a potential coordinated response between transcriptional and metabolic networks during degeneration, though the causal regulatory relationships cannot be inferred from correlation alone. An integrated correlation analysis of metabolomics and transcriptomics identified 20 differentially expressed genes that show highly significant correlations with 15 differential metabolites. The results revealed that the absolute correlation coefficients of most gene–metabolite pairs exceeded 0.99 (for example, the correlation coefficient between Pst134EA_032859 and Sativic acid was −0.9987), and the *p*-values were all below 1 × 10^−5^, indicating extremely strong statistical significance. In terms of regulatory direction, up-regulated genes (such as Pst134EA_032859 and Pst134EA_004947) generally showed negative correlations with decreased metabolite levels, while down-regulated genes (such as Pst134EA_027980 and Pst134EA_000601) mostly exhibited positive correlations with decreased metabolite levels. The metabolite types involved include organic acids (e.g., Hydroxysuberic acid), lipids, alkaloids, and terpenoids, among others. (Supplementary Figure S4).

### 3.4. q-PCR Validation of Differentially Expressed Genes

**Figure 8:**
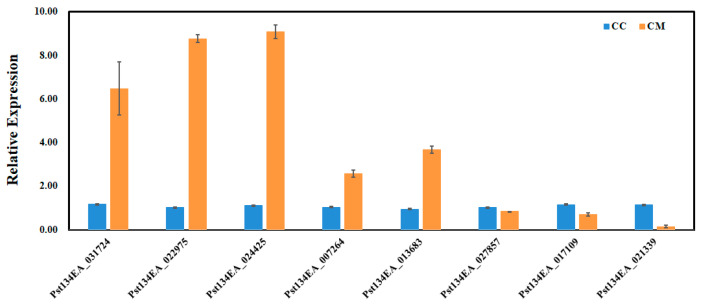
qRT-PCR analyses. Relative gene quantification was calculated by comparative 2−ΔΔCT method. actin Pst134EA_028104 (nei) used as control and mean ± SD of data from three biological replicates was plotted. CC: Fresh spore control; CM: Room-temperature degraded spore treatment.

Eight key target genes closely associated with the natural senescence of wheat stripe rust fungus (involved in peroxidase activity, cytochrome c-heme linkage, and signaling receptor activity, etc.) were randomly selected from the enrichment analysis results for qRT-PCR validation. The results showed that the expression patterns of the selected genes in qRT-PCR were similar to the trends in the transcriptome data (Figure 8). This indicates that the qRT-PCR validation results were consistent with those obtained from the transcriptome sequencing.

## 4. Discussion

This study presents the first integrated transcriptomic and metabolomic profiling of molecular changes during natural degeneration in *Puccinia striiformis* f. sp. *tritici* (Pst) urediniospores. Compared to fresh, highly viable spores, those subjected to room-temperature degeneration exhibited extensive and coordinated molecular alterations. A central observation is the close association between spore degeneration and global transcriptional suppression coupled with downregulation of core metabolic activities. Among the 1622 differentially expressed genes (DEGs) identified, 68.1% (1105) were downregulated, with metabolic pathways also displaying an overall inhibitory trend. This pattern suggests that the natural degeneration of Pst urediniospores involves an orderly decline in biological activity rather than random necrosis.

Transcriptomic analysis revealed significant enrichment of DEGs in KEGG pathways including Ribosome, Oxidative phosphorylation, and Longevity regulation. The widespread downregulation of the Ribosome pathway, which involves numerous ribosomal protein-coding genes such as Pst134EA_013245 (encoding 40S ribosomal protein S15a), indicates a marked reduction in cellular protein synthesis capacity. Similar patterns have been reported in various fungi under severe environmental stress or during senescence. For instance, ribosomal gene downregulation was observed in Aspergillus fumigatus following treatment with the antifungal agent fludioxonil, interpreted as a key step in energy conservation or programmed cell death initiation under irreversible damage [23]. GO enrichment analysis further revealed the biological functions of differentially expressed genes (DEGs). In Biological Process (BP), “cellular nitrogen compound metabolic process” and “biosynthetic process” were significantly enriched and predominantly downregulated, indicating inhibition of nitrogen metabolism and related synthesis pathways during natural degeneration, potentially affecting normal fungal growth and development. Relevant studies confirm the crucial role of nitrogen metabolism pathways in fungal resistance to external stress [24]. In Cellular Component (CC), genes related to “non-membrane-bounded organelle” and “cytosol” were significantly downregulated, suggesting impairment of fundamental cellular structures and functions [25]. In Molecular Function (MF), downregulation of “nucleic acid binding” and “RNA binding” genes was prominent, potentially affecting genetic information processing and stress response capabilities [26,27]. KEGG pathway analysis showed that DEGs were mainly enriched in ribosome, oxidative phosphorylation, and longevity-regulating pathways. The downregulation of these pathways collectively indicates systemic suppression of energy metabolism and biosynthesis [28,29].

Metabolomic analysis revealed that lipids and lipid-like molecules, along with organic acids and derivatives, constituted the major classes of differential metabolites in degenerated spores. This finding is not coincidental. Lipids are not only major components of cell membranes but also crucial energy storage forms and signaling molecules. Lipid metabolism often undergoes reprogramming in fungi responding to environmental stress. For example, studies reported that lipids and lipid-like molecules also represented the highest proportion of differential metabolites when Cryptococcus neoformans was treated with magnolol [30], suggesting that alterations in lipid metabolism are a common feature of stress responses across fungal species. The study found that organic acids and lipids are enriched during the growth and development of Cordyceps militaris [31]. The high proportion of these metabolites in degenerated Pst spores in this study suggests their potential important roles in the degeneration process. Differential metabolites were significantly enriched in metabolic pathways, ABC transporters, and amino acid biosynthesis. The downregulation of amino acid biosynthesis aligns with the suppression of metabolic pathways, indicating an overall reduction in metabolic activity [32]. Changes in ABC transporters may be involved in maintaining cellular homeostasis [33].

Integrative analysis revealed a prevalent negative correlation pattern between gene expression levels and metabolite relative abundance. This statistical association may have multi-layered biological implications. On one hand, it may reflect the decoupling of anabolism (dependent on gene expression and protein synthesis) from catabolism (or passive metabolite accumulation) under the energy- and synthesis-limited state of degeneration. For instance, the downregulation of amino acid biosynthesis pathway genes, coupled with the accumulation of certain amino acids or their derivatives, might arise from a temporary rate of protein degradation exceeding that of synthesis. On the other hand, this widespread negative correlation also suggests a potential global transcriptional response to metabolic state. However, it must be emphasized that correlation analysis per se cannot establish causality or directionality of regulation. This association pattern is descriptive, and the precise regulatory mechanisms behind it—for example, whether metabolite accumulation feedback inhibits gene expression or downregulation of gene expression leads to metabolite homeostasis disruption—await future elucidation through time-series experiments or genetic interventions. Notably, the pathway “Biosynthesis of plant secondary metabolites” was also co-enriched in the integrative analysis. Although purification steps were implemented during sample preparation, this signal may still indicate the presence of trace amounts of wheat host residues. This reflects an inherent challenge in studying obligate biotrophic pathogens. When interpreting the core results, we have taken this background into full consideration and maintain that the changes in core pathways predominantly driven by the Pst spores themselves (e.g., Ribosome and Oxidative phosphorylation) remain the most significant and biologically meaningful findings.

Several limitations merit acknowledgment. Sample purity represents a persistent challenge; despite purification protocols and bioinformatic assessment indicating wheat-derived reads below 5%, obtaining absolutely pure obligate biotrophic fungal spores remains technically difficult. The experimental design comparing only “fresh” and “degenerated” endpoints cannot capture dynamic progression of degeneration. Most importantly, gene–metabolite associations identified through integrative analysis represent statistical correlations that do not imply causal relationships or regulatory hierarchies. Future investigations should prioritize (1) implementing advanced purification methodologies like density gradient centrifugation combined with deep sequencing to minimize and characterize host background; (2) employing time-series sampling integrated with phenotypic metrics such as spore germination rates and membrane integrity to dynamically characterize molecular progression during degeneration; (3) applying genetic manipulation systems to functionally validate key downregulated genes identified here—particularly ribosomal protein genes and oxidative phosphorylation complex subunits—to define their specific roles in spore viability maintenance. Such approaches would advance understanding of regulatory networks governing Pst spore degeneration and potentially inform development of novel control strategies targeting pathogen survival and transmission.

## 5. Conclusions

This integrated transcriptomic and metabolomic analysis provides systematic molecular evidence characterizing the natural degeneration process of *Puccinia striiformis* f. sp. *tritici* urediniospores. The findings demonstrate that spore degeneration is associated with widespread transcriptional downregulation, particularly affecting essential biological pathways including ribosome biogenesis, oxidative phosphorylation, and nitrogen metabolism. Concurrent metabolic profiling reveals significant alterations in lipid and organic acid metabolism, alongside changes in amino acid biosynthesis and transport activities. The coordinated suppression observed across multiple biological systems suggests that spore degeneration represents more than passive cellular deterioration, potentially involving regulated molecular responses to viability loss. The identification of these characteristic molecular signatures advances our understanding of Pst spore biology and provides a foundation for future investigations into mechanisms underlying spore longevity and infectivity. While these findings offer valuable insights, they primarily establish correlative relationships rather than mechanistic explanations. Further research employing time-course experiments, genetic manipulations, and purified spore preparations will be necessary to validate specific regulatory mechanisms and causal relationships identified in this study. The molecular framework established here may inform the development of novel strategies targeting spore viability and persistence, potentially contributing to improved management of wheat stripe rust.

## Figures and Tables

**Table 1:** Summary statistics of clean data from the transcriptomes.

Sample	Raw Reads No	Raw Bases (bp)	Q30 (bp)	GC (%)	N (%)	Q20 (%)	Q30 (%)
CC1	41309910	6,237,796,410	5,972,907,598	47.62	0.111098	98.89	95.75
CC2	42133822	6,362,207,122	6,088,298,822	47.79	0.111401	98.88	95.69
CC3	39513828	5,966,588,028	5,715,005,237	47.6	0.11029	98.89	95.78
CM1	44659164	6,743,533,764	6,467,430,803	46.93	0.110769	98.92	95.91
CM2	43321702	6,541,577,002	6,272,484,020	46.88	0.11074	98.93	95.89
CM3	50467950	7,620,660,450	7,298,152,223	46.72	0.110815	98.9	95.77

## Data Availability

The original contributions presented in this study are included in the article/Supplementary Materials. Further inquiries can be directed to the corresponding author.

## Supplementary Materials

The following supporting information can be downloaded at: https://www.mdpi.com/article/10.3390/cimb48020169/s1.
