# Supplementary material for: Integrated Transcriptomic and Metabolomic Analysis Reveals Molecular Signatures Associated with Natural Degeneration of Puccinia striiformis f. sp. tritici
Source: Curr Issues Mol Biol. 2026 Feb 2;48(2):169. doi: 10.3390/cimb48020169 (PMC12939390; doi:10.3390/cimb48020169)
Supplement: Supplementary file 1 [file cimb-48-00169-s001.zip › Supplementary Table S1.pdf]

Supplementary Table S1. Primers used in qRT-PCR validation.

Primer name	Primer sequence 5'-3'
Pst134EA_031724-F	GCGAAGAATCAAGAATAAG
Pst134EA_031724-R	TCCATTGCCATATTACAG
Pst134EA_022975-F	ATGTTAGACCTGCGATAGAC
Pst134EA_022975-R	AGTAGTAGTGTTAGTGTTAGTGAA
Pst134EA_024425-F	TGACGGTATCAATTATCCA
Pst134EA_024425-R	TCTATCTTCCAACGCAAT
Pst134EA_007264-F	GCTACCGCTAGAGTCTTG
Pst134EA_007264-R	GTGTGGCTTCATCTTGGA
Pst134EA_013683-F	TTCATTGCGAGTATTCTG
Pst134EA_013683-R	TATTCTTGTGTCTGTCTTG
Pst134EA_027857-F	AACCTATTCGTCATCAAG
Pst134EA_027857-R	TGTAGTAGTAGAACTTCCA
Pst134EA_017109-F	CACATTGATTCTCATTGC
Pst134EA_017109-R	CACATAAGACAACTGGAA
Pst134EA_021339-F	TTGGAGTCATTACGATACG
Pst134EA_021339-R	CGGAGTGAGTGGTATTATC
actin Pst134EA_028104(nei)-F	ACTGGTATCGTCTTGGAT
actin Pst134EA_028104(nei)-R	CATGAGGTAGAGCGTAAC
