# Supplementary figures and images for: Integrated Transcriptomic and Metabolomic Analysis Reveals Molecular Signatures Associated with Natural Degeneration of Puccinia striiformis f. sp. tritici
Source: Curr Issues Mol Biol. 2026 Feb 2;48(2):169. doi: 10.3390/cimb48020169 (PMC12939390; doi:10.3390/cimb48020169)

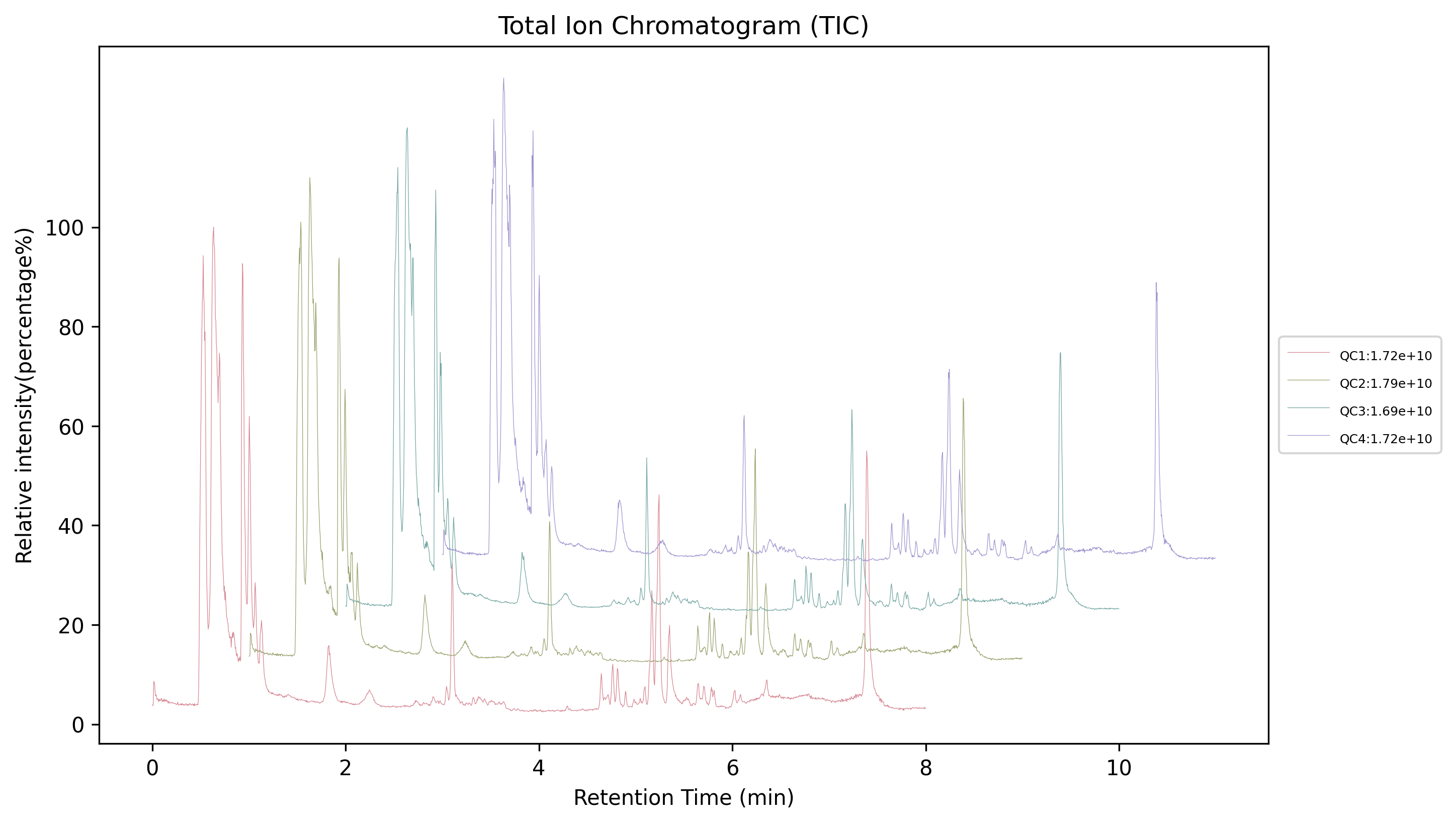

Supplement: Supplementary file 1 [file cimb-48-00169-s001.zip › Supplementary Figure S1.png]

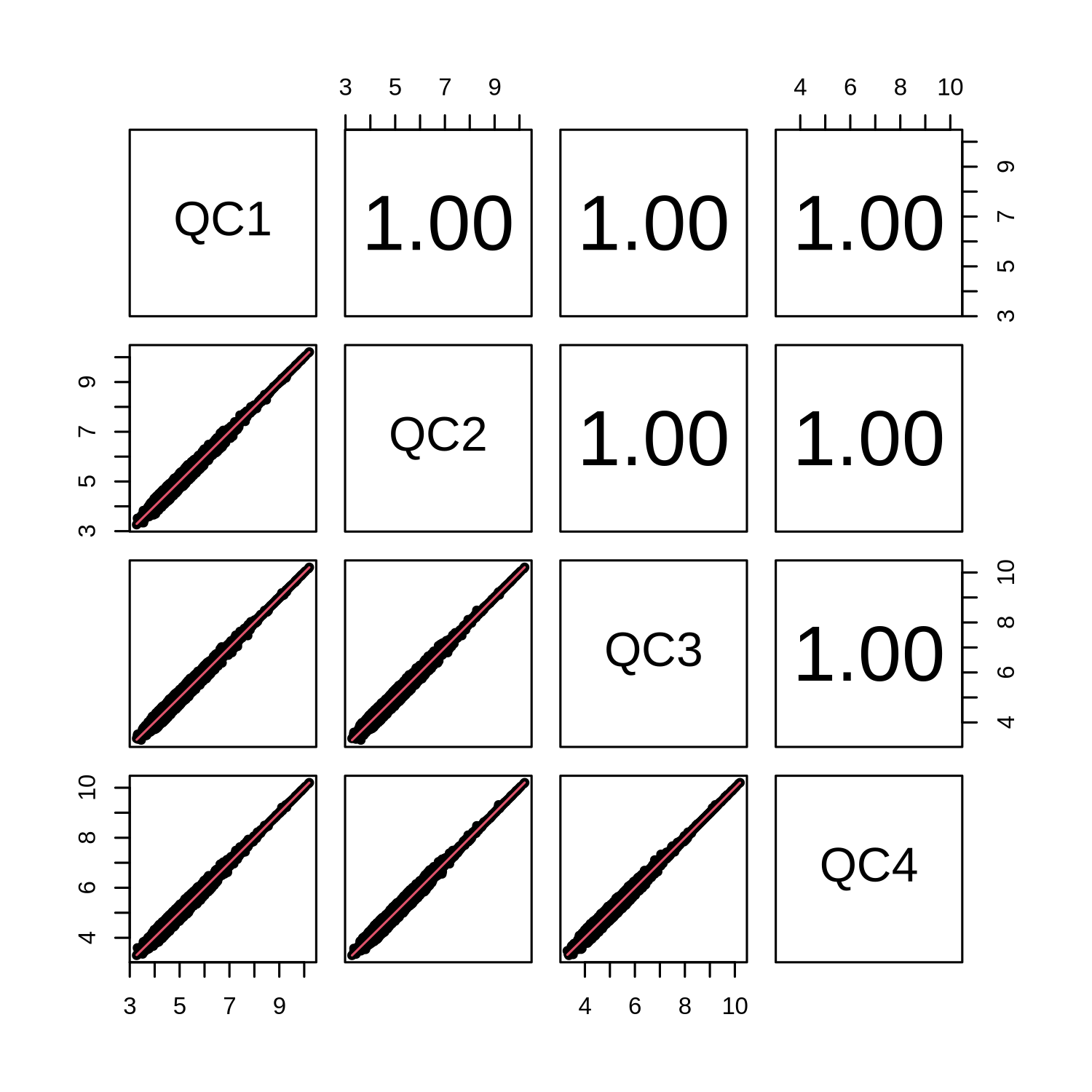

Supplement: Supplementary file 1 [file cimb-48-00169-s001.zip › Supplementary Figure S2.png]

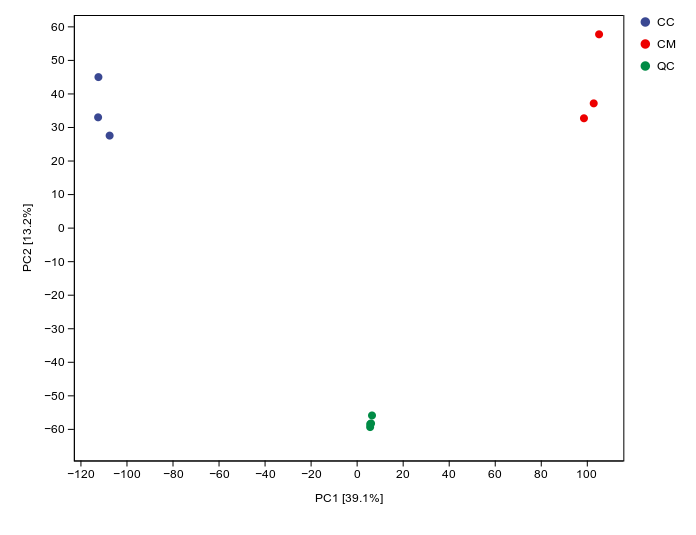

Supplement: Supplementary file 1 [file cimb-48-00169-s001.zip › Supplementary Figure S3.png]

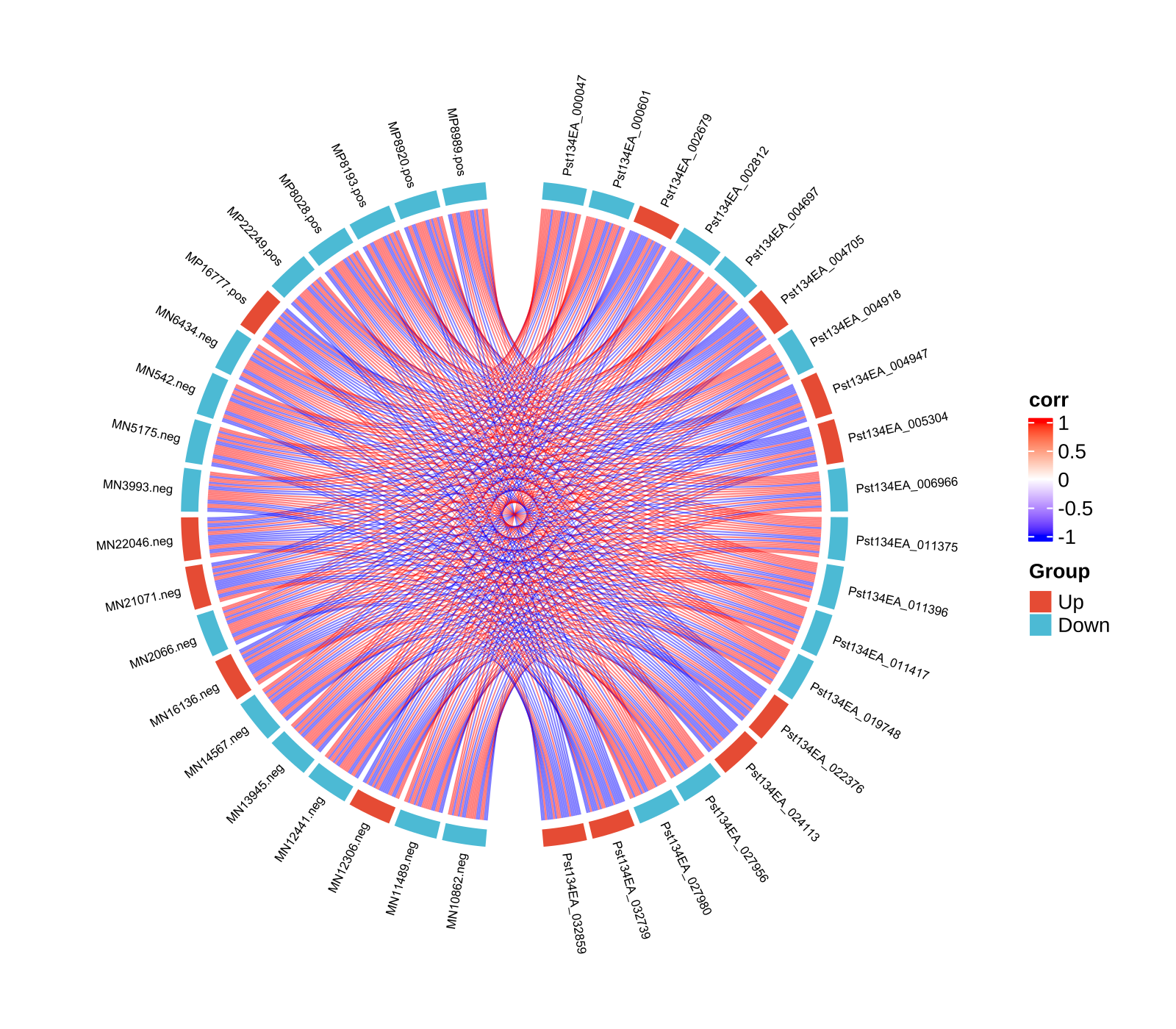

Supplement: Supplementary file 1 [file cimb-48-00169-s001.zip › Supplementary Figure S4.png]
